# The effect of religiosity during childhood and adolescence on drug consumption patterns in adults addicted to crack cocaine

**DOI:** 10.1192/bjo.2018.25

**Published:** 2018-08-02

**Authors:** Alexandre Rezende-Pinto, Alexander Moreira-Almeida, Marcelo Ribeiro, Ronaldo Laranjeira, Homero Vallada

**Affiliations:** Assistant Professor, NUPES – Research Center in Spirituality and Health, School of Medicine, Universidade Federal de Juiz de Fora, Brazil; Associate Professor, NUPES – Research Center in Spirituality and Health, School of Medicine, Universidade Federal de Juiz de Fora, Brazil; National Institute of Public Policy for Alcohol and Other Drugs, Department of Psychiatry, Universidade Federal de São Paulo, Brazil; Professor, National Institute of Public Policy for Alcohol and Other Drugs, Department of Psychiatry, Universidade Federal de São Paulo, Brazil; Associate Professor, Institute and Department of Psychiatry, Faculty of Medicine, Universidade de São Paulo (LIM-23), Brazil.

**Keywords:** Religion, spirituality, cocaine-related disorders, crack cocaine, crime, sexual behaviour, mental disorders, adolescent

## Abstract

**Background:**

Although many studies suggest that religiosity is a protective factor against drug use, there is little information on its effect on drug consumption patterns of those who do use drugs.

**Aims:**

We aimed to examine if there is any relationship between religiosity during childhood and adolescence, and drug consumption in adult crack users.

**Method:**

We performed a cross-sectional study of adults addicted to crack cocaine. Logistic regression analysis was used to estimate the odds ratio for the association between religious history in the age groups 8–11, 12–14 and 15–17 years and outcome variables.

**Results:**

From a total of 531 respondents, religious involvement during childhood and adolescence was correlated to less frequent onset of drug consumption before 18 years (odds ratio 0.95, 95% CI 0.92–0.98) and less craving (odds ratio 0.95, 95% CI 0.91–0.99), mainly between the ages of 15 and 17 years.

**Conclusions:**

Religiosity provides some protection against drug consumption patterns in crack cocaine addicts.

**Declaration of interest:**

None.

## Cocaine use and abuse in the world

Use, misuse and drug addiction has become a public health problem worldwide. It is estimated that a total of 246 million individuals aged between 15 and 64 years used an illicit drug in 2013.^1^ Although there has been a global reduction in cocaine consumption,^1^^,^^2^ an increase from 0.7% in 2010 to 1.2% in 2012 in annual use prevalence has been estimated for South America, largely driven by increasing use in Brazil, especially in the smoked form (crack cocaine).^1^ According to estimates from a recent national survey, in Brazil there are about 2 million individuals who have used crack cocaine at least once in their lives, accounting for 1.5% of adults and 0.8% of adolescents. Brazil has become the second largest market for cocaine in the world, behind only the United States.^3^

The profile of crack cocaine users indicates that most of them are young single males aged under 30 years, of low socioeconomic class, with low education levels and no formal employment.^4^ Crack cocaine users frequently engage in theft and activities related to drug trafficking, and are therefore more likely to be victims of murder.^5^ A cohort study conducted with 131 crack cocaine addicts admitted to rehabilitation in São Paulo showed that 18.5% had died within 5 years of discharge, with murder being the most frequent cause.^6^ Other problems among crack cocaine users are sexual risk-seeking behaviour and HIV infection.^5^^,^^7^

Data indicate that about half of Brazilian cocaine users first used the drug before 18 years of age.^3^ The marked increase in prevalence rates from 13 to 18 years of age characterises this period as key in the development of disorders caused by this type of substance.^8^ The early use of drugs is associated with multiple demographic, social, psychological, personality and family factors.^9^^,^^10^ The onset and patterns of drug use are strongly influenced by environmental, social and family factors during adolescence, with genetic factors becoming progressively more important until the beginning of adulthood.^11^ Among these factors, in recent decades, religiosity and spirituality have emerged as a protective factor against drug use.^12^ A review examining the relationship between religiosity and spirituality and drug misuse found that, of a total of 185 studies, 84% reported less misuse of drugs among the most religious individuals. More than 70% of those studies had been conducted with young individuals (adolescents, college students and young adults).^13^ Although it has already been shown that greater levels of religiosity and spirituality are associated with less drug use and misuse, the mechanisms are not yet clear.

## Childhood religiosity and drug use in adults

There has been a growing interest in assessing how religiosity during childhood and adolescence can influence mental health in later years; however, studies are still scarce. A prospective study monitored 114 children of depressed and non-depressed parents for 10 years. The children of depressed parents who reported high importance of religion or spirituality had about one-tenth the risk of experiencing major depression compared with others in the high-risk group over the 10-year follow-up period.^14^ With respect to the influence of religious involvement during childhood and adolescence and subsequent use of drugs, a longitudinal study conducted with adolescents over 1 year found that around 65% of them had not experienced changes in religiosity. However, those who had a decline in their religiosity exhibited greater rates of drug use.^15^ A cross-sectional study showed that heavy use of at least one illicit drug was greater (odds ratio 1.67) among adolescent students who had no religious upbringing during childhood.^16^ Nevertheless, few studies provide data on religiosity in early life and its effect on drug use patterns and other risk-seeking behaviours of drug users in later life. Similarly, although there are many studies showing the protective effect of religiosity and spirituality against the development of drug addiction, there is a lack of studies addressing the religious history of drug dependents and how this history might have influenced the development of drug dependence.

The goal of this study was to assess the effect of religious involvement during childhood and adolescence in a population of adults addicted to crack cocaine who were admitted to treatment in therapeutic communities on consumption patterns, involvement in criminality and sexual risk-seeking behaviour; quality of life and psychiatric comorbidities. In addition, we assessed the possible mechanisms involved in the relationship between religiosity and spirituality and crack cocaine use patterns. The therapeutic communities treatment model in the field of chemical dependency is available in most countries,^17^ with spirituality an essential factor in its approach.^18^

## Method

### Sample and procedures

This cross-sectional study is part of a large project assessing the history of crack cocaine consumption of addicts from seven Brazilian states from different regions, approved by the local research ethics committees (appoval number from the Research Ethics Committee, Universidade Federal de São Paulo: CAAE 00559212.1.0000.5505). A total of 20 therapeutic communities linked to the Brazilian Federation of Therapeutic Communities (FEBRACT; http://www.febract.org.br) agreed to participate in the study. All crack cocaine users aged over 18 years of age who had started treatment in the therapeutic communities between September 2012 and September 2013 were invited to participate in the study. All respondents signed an informed consent form.

### Assessment and measurement

A very detailed structured questionnaire was developed by M.R. and R.L., integrating parts or the whole of the following questionnaires: the Drug Abuse Treatment Outcome Study, the Maudsley Addiction Profile, the Medical College of Virginia Stress and Coping Study and the Crack Cocaine Craving Questionnaire-Brief (CCQ-Brief), as well as a number of scales and tests, including the Duke University Religion Index (DUREL), the World Health Organization Quality of Life-Brief instrument and the Mini International Neuropsychiatric Interview (MINI). In total, the whole assessment presented more than 900 variables. A more detailed description of the whole assessment can be seen at http://www.researchgate.net/publication/283153684. Health professionals were trained to administer the application, which was undertaken during the first days of the users' admission to the therapeutic communities.

#### Control variables

Demographic control variables were age, gender, living with a partner and education.

#### Predictor variables

##### Current religiosity and spirituality

To assess the patients' current religiosity and spirituality, we used the DUREL, a self-completed five-item scale.^19^ Lucchetti *et al* validated a Portuguese version.^20^ It evaluates three dimensions of religiosity and spirituality: organisational religiosity, non-organisational religiosity and intrinsic religiosity.

Organisational religiosity was measured by the frequency of attendance at churches and temples services (the six possible answers were dichotomised between one or more and less than one time per week). Non-organisational religiosity was evaluated by the time dedicated to prayer, meditation and reading the Bible or other religious texts (dichotomised between more than once and less than once per day). Intrinsic religiosity analyses to what extent religious beliefs influence the life of the respondents (a score ≥12 is considered positive).

##### Past religiosity and spirituality

The questions to assess past religiosity and spirituality were originally extracted from the Medical College of Virginia Stress and Coping Study questionnaire and repeated for three different periods of the life of each participant. The age periods were 8–11 years (period I), 12–14 years (period II) and 15–17 years (period III). A brief summary of the questions and respective scores are presented below.

To assess religious frequency: ‘When you were between x and y years old, did you attend any religious services, such as masses, Evangelical cults, spiritualist sessions and/or Afro-Brazilian religions?’ The scores ranged from 0 (never) to 5 (more than once a week). To assess religious participation: ‘When you were between x and y years old, how often did you participate in activities relating to religion, such as meetings, youth groups, social and charitable work?’ The scores ranged from 0 (never) to 3 (often). To assess religious history, we examined frequency of attendance and of participation in religious activities for each of the three different age periods. The global religious frequency and the global religious participation scores were obtained by the sum of the religious frequency and religious participation, respectively, in each of the three age groups, and the global religious history score was obtained by the sum of the frequency and participation scores in the three age groups.

#### Outcome variables

##### Severity of crack cocaine consumption

We assessed severity of crack cocaine consumption by the following variables: age at onset of crack cocaine consumption (dichotomised between ≥ 18 years of age and <18 years of age), number of crack cocaine rocks consumed at a single time during the greatest consumption stage (dichotomised between ≥ 10 or <10 crack cocaine rocks). This value was chosen because a recent national Brazilian survey showed that the average consumption in Brazil was 13.42 crack cocaine rocks in a ‘normal day’,^21^ and total craving, assessed by the Brazilian version of the CCQ-Brief,^22^^,^^23^ exhibited a score of 10–70, which was dichotomised between ≥ 28 and <28 points, corresponding to the average and the s.d.

##### Involvement in criminality and sexual risk-seeking behaviour

We assessed involvement in criminality and sexual risk-seeking behaviour by the following variables: having been arrested after 18 years of age, having been earning a living from illegal activities for at least 1 month at any time, having been able to receive drugs in exchange for sex and having had sexual intercourse with >10 different individuals in a single year.

##### Quality of life

Quality of life was assessed by the World Health Organization Quality of Life-Brief instrument, which comprises 26 questions divided into four domains (physical, psychological, social relationships and environmental).^24^ Fleck *et al* validated a Portuguese version.^25^

##### Psychiatric comorbidities

Psychiatric comorbidities were assessed by examining current depression and suicide risk (suicidal ideation and suicide attempt) in the previous 30 days, assessed by the MINI.^26^ A Portuguese version was validated by Amorim.^27^

#### Mediator variables

The following variables were used as possible mediators of the effect of religious involvement during childhood and adolescence on outcome variables in the same age groups described above. Social environment: ‘How often did you participate in regular sports activities (in and out of school) or extracurricular activities (courses, cultural activities)?’ Family ties: ‘How often did you undergo unpleasant situations or disagreements, fights or conflicts with your mother (caregiver) or father (caregiver)’; and ‘how was your relationship with your mother (caregiver) or father (caregiver)?’

### Statistical analyses

All analyses were conducted with the Stata 11 statistical software package. Bivariate analysis (Student's *t*-test for continuous variables and χ^2^-test for categorical variables) was used to calculate differences in outcome variables and the dimensions of current religiosity of individuals (organisational religiosity, non-organisational religiosity and intrinsic religiosity). Logistic regression analysis was used to estimate the odds ratio, adjusted for age, gender, living with a partner and education, with a 95% confidence interval. Linear regression analysis was used to evaluate the relationship between religious history and quality of life.

A Sobel–Goodman mediation test in Stata 11 to estimate the mediator effect of social environment and family ties on the association between the predictor variables and outcome was used. In general terms, it can be stated that mediation occurs when (a) the predictor variable significantly affects the mediator, (b) the predictor variable significantly affects the outcome in the absence of the mediator, (c) the mediator has a significant effect on the outcome variable and (d) the effect of the predictor variable on the outcome variable decreases after the addition of the mediator in the model.

## Results

A total of 642 individuals answered the questionnaire, but our final sample was composed of 531 heavy adult users of crack cocaine admitted to treatment in therapeutic communities. There were 111 individuals who did not complete all the variables of interest for our analysis. The majority were single males (89.4%) with an average age of 30.9 years, a low educational level and high to moderate levels of religious beliefs and frequency of attendance at religious services. With respect to the severity of crack cocaine consumption, the average age at onset of use was 21.6 years (s.d. 7.7), 78.9% used >10 crack cocaine rocks at a single time during the greatest consumption stage and had an average score of 18.4 (s.d. 9.6) on the craving scale of the CCQ-Brief. Almost half had been arrested after 18 years of age and had earned a living from illicit activities for at least 30 days at some period in their life. Almost one-third had received drugs in exchange for sex, and 55.7% had >10 sexual partners in a single year. Regarding psychiatric comorbidities, 14.6% met the criteria for depression at the time of admission according to the MINI, and 57.2% had been at risk of death by suicide during the previous 30 days (Table 1).

**Table 1:** Characteristics of the sample during admission

Variable	Total sample *N* = 531	DUREL (current religiosity and spirituality)					
		Organisational religiosity		Non-organisational religiosity		Intrinsic religiosity	
		No *N* = 349 (65.7%)	Yes *N* = 182 (34.3%)	No *N* = 287 (54.1%)	Yes *N* = 244 (45.9%)	No *N* = 198 (37.3%)	Yes *N* = 333 (62.7%)
Demographic variable							
Gender, *n* (%)							
Female	56 (10.6)	32 (9.2)	24 (13.2)	24 (8.4)	32 (13.1)	18 (9.1)	38 (7.4)
Male	475 (89.4)	317 (90.8)	158 (86.8)	263 (91.6)	212 (86.9)	180 (90.1)	295 (88.6)
Age, average (s.d.)	30.9 (7.7)	30.9 (7.9)	30.9 (7.3)	**30.2 (7.6)**	**31.7 (7.8)**	30.4 (8.2)	31.2 (7.4)
Living with a partner, *n* (%)							
No	411 (78.4)	277 (80.1)	134 (75.3)	218 (76.8)	193 (80.4)	155 (79.5)	256 (77.8)
Yes	113 (21.6)	69 (19.9)	44 (24.7)	66 (23.2)	47 (19.6)	40 (20.5)	73 (22.2)
Education, *n* (%)							
Primary education (incomplete)	126 (24.0)	92 (26.7)	34 (18.9)	71 (25.1)	55 (22.7)	35 (17.9)	91 (27.6)
Primary education (complete)	69 (13.1)	40 (11.6)	29 (16.1)	38 (13.4)	31 (12.8)	29 (14.9)	40 (12.1)
Secondary education (incomplete)	121 (23.0)	83 (24.1)	38 (21.1)	70 (24.7)	51 (21.1)	49 (25.1)	72 (21.8)
Secondary education (complete)	209 (39.8)	130 (37.6)	79 (43.9)	104 (36.8)	105 (43.4)	82 (41.1)	127 (38.5)
Severity of crack cocaine consumption							
Age at onset, average (s.d.)	21.6 (7.1)	21.2 (7.2)	22.3 (6.9)	**20.8 (6.4)**	**22.6 (7.8)**	20.9 (6.9)	22.0 (7.3)
Greatest consumption (rocks), *n* (%)							
1–10	111 (21.1)	74 (21.3)	37 (20.8)	65 (23.0)	46 (18.9)	35 (17.9)	76 (23.0)
>10	415 (78.9)	274 (78.7)	141 (79.2)	218 (77.0)	197 (81.1)	161 (82.1)	254 (77.0)
Total craving, average (s.d.)	18.4 (9.6)	18.7 (9.6)	17.7 (9.4)	**19.3 (10.2)**	**17.3 (8.6)**	19.7 (10.3)	17.6 (9.0)
Criminality							
Imprisonment >18 years of age, *n* (%)							
No	295 (55.6)	**178 (51.0)**	**117 (64.3)**	153 (53.3)	142 (58.2)	106 (53.5)	189 (56.8)
Yes	236 (44.4)	**171 (49.0)**	**65 (35.7)**	134 (46.7)	102 (41.8)	92 (46.5)	144 (43.2)
Living off of crime, *n* (%)							
No	288 (54.3)	183 (52.4)	105 (58.0)	148 (51.6)	140 (57.6)	109 (55.1)	179 (53.9)
Yes	242 (45.7)	166 (47.6)	76 (42.0)	139 (48.4)	103 (42.4)	89 (44.9)	153 (46.1)
Sexual risk-seeking behaviour							
Sex for drugs, *n* (%)							
No	373 (70.9)	246 (71.1)	127 (70.6)	197 (69.4)	176 (72.7)	135 (69.6)	238 (71.7)
Yes	153 (29.1)	100 (28.9)	53 (29.4)	87 (30.6)	66 (27.3)	59 (30.4)	94 (28.3)
Sex with >10 individuals in a year, *n* (%)							
No	233 (44.3)	158 (45.9)	75 (41.2)	127 (44.9)	106 (43.6)	85 (43.8)	148 (44.6)
Yes	293 (55.7)	186 (54.1)	107 (58.8)	156 (55.1)	137 (56.4)	109 (56.2)	184 (55.4)
Quality of life							
WHOQOL-Brief, average (s.d.)							
Physical health	75.4 (16.4)	75.1 (16.9)	75.8 (15.5)	75.9 (16.9)	74.8 (15.8)	**73.2 (16.6)**	**76.6 (16.2)**
Psychological health	65.3 (17.2)	**64.1 (18.0)**	**67.7 (15.3)**	**63.9 (18.4)**	**67.0 (15.5)**	**61.6 (17.8)**	**67.5 (16.5)**
Social relationships	60.3 (20.1)	59.8 (20.9)	61.3 (18.5)	60.5 (20.6)	60.1 (19.6)	**57.6 (20.7)**	**61.9 (19.7)**
Environmental health	61.6 (15.7)	61.1 (16.3)	62.8 (14.5)	61.0 (16.7)	62.4 (14.4)	**59.0 (16.6)**	**63.2 (15.0)**
Psychiatric comorbidities							
Depression, *n* (%)							
No	450 (85.4)	297 (85.3)	153 (85.5)	242 (84.6)	208 (86.3)	171 (87.7)	279 (84.0)
Yes	77 (14.6)	51 (14.7)	26 (14.5)	44 (15.4)	33 (13.7)	24 (12.3)	53 (16.0)
Suicide, *n* (%)							
No	221 (42.8)	147 (43.0)	74 (42.3)	128 (45.2)	93 (39.7)	76 (39.4)	145 (44.8)
Yes	296 (57.2)	195 (57.0)	101 (57.7)	155 (54.8)	141 (60.3)	117 (60.6)	179 (55.2)
Social environment and family ties							
Social environment, average (s.d.)	10.1 (4.6)	**9.7 (4.5)**	**10.8 (4.6)**	**9.7 (4.6)**	**10.6 (4.5)**	**9.5 (4.4)**	**10.4 (4.7)**
Family ties, average (s.d.)	34.6 (11.9)	34.0 (12.1)	35.8 (11.5)	34.2 (11.3)	35.1 (12.6)	34.6 (11.9)	34.6 (11.9)

Numbers in bold indicate *P* < 0.05 (bivariate analysis).

DUREL, Duke University Religion Index; WHOQOL-Brief, World Health Organization Quality of Life-Brief instrument.

As shown in Table 1, the bivariate analysis of the consumption parameters, risk-seeking behaviours and current religiosity showed that non-organisational religiosity was associated with a mean age >1.5 years at the time of admission, later consumption onset and less craving. Current frequency of attendance at religious services (organisational religiosity) was associated with a marked decrease in imprisonment after 18 years of age (from 49 to 35.7%). Intrinsic religiosity was associated with less craving and better quality of life in the four dimensions. Quality of life in the psychological domain was greater in the three religiosity dimensions.

Religious history (i.e. past religious involvement during childhood and adolescence) is analysed in Table 2. Global religious frequency (attendance at religious services) was associated with higher educational level, later onset of and lower intensity of crack consumption, less imprisonment as an adult and less likelihood of lifelong criminal activities. Religious history (frequency and participation) was associated with better quality of life in the domain of psychological health. Regarding physical health, the results were contradictory, as global religious history score was associated with better physical health, whereas global religious frequency score was associated with worse physical health. Higher global religious participation scores were associated with a younger age group (on average 1.4 years younger).

**Table 2:** Distribution of religious history variables

Variable	Total sample *N* = 531	Religious history scores					
		Global religious history		Global religious frequency		Global religious participation	
		<14 *N* = 355 −71.60%	≥14 *N* = 141 −28.40%	<11 *N* = 363 −71.70%	≥11 *N* = 143 −28.30%	<4 *N* = 352 −68.50%	≥4 *N* = 162 −31.50%
Demographic variable							
Gender, *n* (%)							
Female	56 (10.6)	39 (11.0)	11 (7.8)	43 (11.8)	10 (7.0)	36 (10.2)	14 (8.6)
Male	475 (89.4)	316 (89.0)	130 (92.2)	320 (88.2)	133 (93.0)	316 (89.8)	148 (91.4)
Age, average (s.d.)	30.9 (7.7)	31.3 (7.8)	30.0 (7.4)	30.8 (7.7)	31.2 (7.8)	**31.3 (7.8)**	**29.9 (7.2)**
Living with a partner, *n* (%)							
No	411 (78.4)	277 (78.5)	109 (77.8)	286 (79.2)	106 (74.6)	280 (80.0)	123 (76.4)
Yes	113 (21.6)	76 (21.5)	31 (22.2)	75 (20.8)	36 (25.4)	70 (20.0)	38 (23.6)
Education, *n* (%)							
Primary education (incomplete)	126 (24.0)	87 (24.6)	27 (19.1)	**91 (25.2)**	**27 (18.9)**	92 (26.3)	29 (17.9)
Primary education (complete)	69 (13.1)	46 (13.0)	18 (12.8)	**51 (14.1)**	**15 (10.5)**	45 (12.9)	22 (13.6)
Secondary education (incomplete)	121 (23.0)	90 (25.5)	28 (19.9)	**92 (25.5)**	**28 (19.6)**	83 (23.7)	36 (22.2)
Secondary education (complete)	209 (39.8)	130 (36.9)	68 (48.2)	**127 (35.2)**	**73 (51.0)**	130 (37.1)	75 (46.3)
Severity of crack cocaine consumption							
Age at onset, average (s.d.)	21.6 (7.1)	21.4 (6.9)	22.0 (7.2)	**21.0 (6.7)**	**23.0 (7.5)**	21.5 (6.9)	21.7 (7.2)
Greatest consumption (rocks), *n* (%)							
1–10	111 (21.1)	69 (19.6)	32 (22.9)	**61 (17.0)**	**41 (28.7)**	73 (21.0)	33 (20.5)
>10	415 (78.0)	283 (80.4)	108 (77.1)	**298 (83.0)**	**102 (71.3)**	275 (79.0)	128 (79.5)
Total craving, average (s.d.)	18.4 (9.6)	18.7 (10.0)	17.8 (8.6)	18.7 (9.8)	17.6 (8.9)	18.8 (10.2)	17.6 (8.2)
Criminality Imprisonment >18 years of age, *n* (%)							
No	295 (55.6)	193 (54.4)	85 (60.3)	**189 (52.1)**	**92 (64.3)**	186 (52.8)	100 (61.7)
Yes	236 (44.4)	162 (45.6)	56 (39.7)	**174 (47.9)**	**51 (35.7)**	166 (47.2)	62 (38.3)
Living off of crime, *n* (%)							
No	288 (54.3)	188 (53.1)	85 (60.3)	**185 (51.1)**	**92 (64.3)**	189 (53.8)	90 (55.6)
Yes	242 (45.7)	166 (46.9)	56 (39.7)	**177 (48.9)**	**51 (35.7)**	162 (46.2)	72 (44.4)
Sexual risk-seeking behaviour							
Sex for drugs, *n* (%)							
No	373 (70.9)	248 (70.7)	99 (70.2)	252 (70.2)	102 (71.3)	245 (70.2)	115 (71.4)
Yes	153 (29.1)	103 (29.3)	42 (29.8)	107 (29.8)	41 (28.7)	104 (29.8)	46 (28.6)
Sex with >10 individuals in a year, *n* (%)							
No	233 (44.3)	150 (42.6)	62 (44.3)	151 (41.8)	69 (48.9)	146 (41.8)	75 (46.6)
Yes	293 (55.7)	202 (57.4)	78 (55.7)	210 (58.2)	72 (51.1)	203 (58.2)	86 (53.4)
Quality of life WHOQOL-Brief, average (s.d.)							
Physical health	75.4 (16.4)	**74.4 (16.6)**	**78.2 (15.6)**	**74.0 (17.0)**	**72.8 (15.1)**	74.8 (15.8)	77.0 (17.2)
Psychological health	65.3 (17.2)	**64.1 (17.7)**	**68.6 (15.0)**	**64.0 (17.9)**	**68.4 (15.1)**	**64.4 (17.3)**	**67.6 (16.6)**
Social relationships	60.3 (20.1)	59.9 (20.8)	61.4 (18.4)	59.2 (21.0)	62.8 (17.7)	60.0 (20.4)	61.0 (20.0)
Environmental health	61.6 (15.7)	61.2 (15.5)	64.1 (15.4)	61.6 (15.8)	62.4 (15.2)	60.9 (15.2)	63.8 (16.4)
Psychiatric comorbidities Depression, *n* (%)							
No	450 (85.4)	303 (86.3)	119 (84.4)	309 (86.1)	121 (84.6)	297 (85.3)	139 (85.8)
Yes	77 (14.6)	48 (13.7)	22 (15.6)	50 (13.9)	22 (15.4)	51 (14.7)	23 (14.2)
Suicide, *n* (%)							
No	221 (42.8)	152 (44.1)	58 (42.0)	155 (43.9)	59 (42.1)	146 (42.9)	69 (43.1)
Yes	296 (57.2)	193 (55.9)	80 (58.0)	198 (56.1)	81 (57.9)	194 (57.1)	91 (56.9)

Numbers in bold indicate *P* < 0.05 (bivariate analysis).

WHOQOL-Brief, World Health Organization Quality of Life-Brief instrument.

Through multivariate analysis (Table 3), which assessed the effect of religiosity and spirituality involvement throughout childhood and adolescence, controlling for sociodemographic variables, we found that the global religious history was associated with less chance of beginning crack consumption before 18 years of age (odds ratio 0.95, 95% CI 0.92–0.98) and less severe craving (odds ratio 0.95, 95% CI 0.91–0.99). The age group 15–17 years exhibited more statistically significant correlations, with less chance of beginning consumption before 18 years of age (odds ratio 0.87, 95% CI 0.80–0.95), consumption of >10 crack cocaine rocks at a single time (odds ratio 0.87, 95% CI 0.80–0.95) and less serious craving (odds ratio 0.86, 95% CI 0.76–0.97).

**Table 3:** Logistic regression between religious history and severity of crack cocaine consumption

Variable (total scores)	Age at onset (before 18 years), odds ratio (95% CI)	Greatest consumption (>10 crack cocaine rocks), odds ratio (95% CI)	Total craving (CCQ-Brief score >28), odds ratio (95% CI)
Global religious history	**0.95 (0.92–0.98)**	0.96 (0.93–1.00)	**0.95 (0.91–0.99)**
Religious history (frequency and participation) at 8–11 years of age	**0.91 (0.84–0.98)**	0.99 (0.91–1.07)	0.95 (0.86–1.04)
Religious history (frequency and participation) at 12–14 years of age	0.94 (0.87–1.01)	0.95 (0.88–1.04)	**0.88 (0.80–0.97)**
Religious history (frequency and participation) at 15–17 years of age	**0.87 (0.80–0.95)**	**0.87 (0.80–0.95)**	**0.86 (0.76–0.97)**
Global religious frequency	**0.92 (0.88–0.97)**	**0.94 (0.89–0.99)**	0.95 (0.90–1.00)
Global religious participation	0.93 (0.86–1.01)	0.98 (0.90–1.06)	**0.85 (0.76–0.95)**
Frequency of attendance at religious services at 8–11 years of age	**0.88 (0.72–0.97)**	0.95 (0.85–1.06)	0.98 (0.86–1.10)
Participation in religious activities at 8–11 years of age	0.90 (0.77–1.07)	1.07 (0.90–1.28)	0.82 (0.66–1.02)
Frequency of attendance at religious services at 12–14 years of age	0.90 (0.81–1.01)	0.93 (0.82–1.04)	**0.85 (0.74–0.98)**
Participation in religious activities at 12–14 years of age	0.93 (0.77–1.12)	0.94 (0.78–1.15)	0.78 (0.60–1.00)
Frequency of attendance at religious services at 15–17 years of age	**0.85 (0.75–0.96)**	**0.83 (0.73–0.93)**	**0.86 (0.74–0.99)**
Participation in religious activities at 15–17 years of age	**0.71 (0.56–0.91)**	0.81 (0.65–1.02)	**0.59 (0.41–0.86)**
Second section: comparison between quartiles			
Global religious history, first × fourth quartile	**0.50 (0.28–0.90)**	0.67 (0.36–1.25)	0.52 (0.26–1.06)
Religious history (frequency and participation) at 8–11 years of age, first × fourth quartile	**0.56 (0.32–0.98)**	0.91 (0.48–1.69)	0.81 (0.43–1.52)
Religious history (frequency and participation) at 12–14 years of age, first × fourth quartile	0.65 (0.38–1.11)	0.81 (0.45–1.48)	**0.42 (0.21–0.84)**
Religious history (frequency and participation) at 15–17 years of age, first × fourth quartile	**0.53 (0.32–0.88)**	**0.46 (0.27–0.76)**	**0.50 (0.26–0.98)**
Global religious frequency, first × fourth quartile	**0.30 (0.16–0.56)**	**0.48 (0.26–0.88)**	0.64 (0.31–1.32)
Global religious participation, first × fourth quartile	0.77 (0.46–1.30)	1.12 (0.66–1.90)	**0.42 (0.22–0.79)**

Controlling for age, gender, living with a partner and education Numbers in bold indicate statistical significance.

CCQ-Brief, Crack Cocaine Craving Questionnaire-Brief.

The global frequency of attendance at religious services (i.e. the sum of the scores in the three age groups) protected against consumption onset before the age of 18 years (odds ratio 0.92, 95% CI 0.88–0.97) and consumption of >10 crack cocaine rocks at a single time (odds ratio 0.94, 95% CI 0.89–0.99). The global religious participation of the three age groups correlated only with less severe craving (odds ratio 0.85, 95% CI 0.76–0.95). The frequency of attendance at religious services between 15 and 17 years of age had inverse correlation with the three markers of crack cocaine consumption severity: onset before 18 years of age (odds ratio 0.85, 95% CI 0.75–0.96), >10 crack cocaine rocks consumed at a single time during the period of greatest consumption (odds ratio 0.83, 95% C 0.73–0.93) and severe craving (odds ratio 0.86, 95% CI 0.74–0.99) (Table 3).

To assess the effect of religious involvement during childhood and adolescence, we compared individuals with greater scores (first quartile) and those with the lowest scores (fourth quartile). We found a very similar pattern of inverse correlations between religious involvement during childhood and adolescence and the severity indicators of crack cocaine use, with the difference that the effect was much greater (odds ratio 0.30–0.56).

There were no statistically significant differences in the logistic regression of the religious history parameters with criminality, sexual risk-seeking behaviour and psychiatric comorbidities (current depression and suicide risk in the previous 30 days).

Table 4 shows the variables of religious involvement during childhood and adolescence correlated with dimensions of quality of life, notably the dimensions of psychological and environmental health. The relationship between religious involvement from 15 to 17 years and lower likelihood of starting crack cocaine consumption before 18 years of age was partially mediated by involvement in social activities (sports and cultural), with a 22% ratio of the total effect. In addition, the quality of relationship with parents in this age group also mediated the effect of religious involvement and onset of crack cocaine consumption, with a 17% ratio of the total effect.

**Table 4:** Linear regression between religious history and quality of life (WHOQOL-Brief)

Variable (total scores)	Physical health, coefficient (95% CI)	Psychological health, coefficient (95% CI)	Social relationships, coefficient (95% CI)	Environmental health, coefficient (95% CI)
Global religious history	0.19 (−0.52 to 0.43)	**0.42 (0.17–0.68)**	0.19 (−0.11 to 0.49)	**0.24 (0.01–0.47)**
Religious history (frequency and participation) at 8–11 years of age	0.34 (−0.21 to 0.90)	**0.81 (0.22–1.40)**	0.40 (−0.28 to 1.09)	**0.73 (0.20–1.26)**
Religious history (frequency and participation) at 12–14 years of age	0.21 (−0.34 to 0.75)	**0.68 (0.10–1.25)**	0.14 (−0.54 to 0.81)	0.41 (−0.11 to 0.93)
Religious history (frequency and participation) at 15–17 years of age	**0.64 (0.62–1.23)**	**1.10 (0.48–1.71)**	0.55 (−0.18 to 1.28)	0.39 (−0.17 to 0.95)
Global religious frequency	**0.35 (0.02–0.68)**	**0.56 (0.21–0.91)**	**0.43 (0.21–0.84)**	0.29 (−0.03 to 0.61)
Global religious participation	0.83 (−0.47 to 0.63)	**0.75 (0.17–1.33)**	−0.05 (−0.74 to 0.64)	**0.54 (0.02–1.07)**

**C**ontrolling for age, gender, living with a partner and education. Numbers in bold indicate statistical significance.

WHOQOL-Brief, World Health Organization Quality of Life-Brief instrument.

## Discussion

The results showed the effect of religiosity in childhood and adolescence on drug consumption patterns in 531 adults dependent on crack cocaine. Religious involvement in childhood and adolescence (8–17 years old) was related to less initiation of consumption before the age of 18 and less current craving. Religious involvement in the range of 15 to 17 years was inversely associated with consumption of >10 rocks at once during the largest consumption period. The relationship between religious involvement from 15 to 17 years and less chance of early crack use before the age of 18 was partially mediated by social activities and relationships with parents. Current religious participation is related to lower craving, less imprisonment and better quality of life, mainly in the psychological domain.

We compared the profile of our sample with a recent national Brazilian survey of crack users.^21^ In our study, we found a greater proportion of male individuals (89.4 *v.* 76.7%), single individuals (78.4 *v.* 60.6%) and those who completed secondary education (39.8 *v.* 16.5%). The average ages were very similar (30.9 *v.* 30.3 years), as were the proportion of individuals who had been arrested after 18 years of age (44.4 *v.* 48.8%). With respect to having received drugs in exchange for sex in the previous 30 days, our study found a proportion of 29.1% *v.* 42.17% in the other study. We also found high rates of psychiatric comorbidities: 14.6% with criteria for current depression and 57.2% with suicide risk in the previous 30 days. A meta-analysis had indicated a strong association between major depression and disorders caused by illicit drug use (odds ratio 3.80, 95% CI 3.02–4.78).^28^ Another meta-analysis had reported a significant association between disorders caused by drug use and suicidal ideation (odds ratio 2.04, 95% CI 1.59–2.50).^29^

Current religiosity and religiosity during childhood and adolescence were associated with various indicators of a less severe crack cocaine addiction, even in a sample of individuals with severe dependence. With respect to current religiosity, although half of those who did not attend religious services had been arrested, the proportion fell to one-third among those who attended the services. A systematic review found that 79% of 102 quantitative studies reported an inverse relationship between religiosity and spirituality and crime and delinquency.^13^ Regnerus and Elder found that religious frequency and importance of religion predicted lower delinquency in adolescents followed for 12 months at the National Longitudinal Study of Adolescent Health (Add Health).^30^ In our study, intrinsic religiosity was associated with a less severe craving and the best quality of life in all domains. A longitudinal study that followed 439 untreated crack users for 2 years showed that, overall, the quality of life of active crack users is inversely proportional to the frequency and severity of drug use.^31^

Associations between different age groups were also observed. In Table 1, a higher current non-organisational religiosity was correlated with older age (on average 1.5 years older) and in Table 2, higher global religious participation score was associated with younger age (on average 1.4 years younger). However, the comparison of the results of Tables 1 and 2 are somewhat contradictory because the associations are in opposite direction (one group is younger and the other older). Nevertheless, the age differences in the other groups (e.g. organisational religiosity and intrinsic religiosity in Table 1 and global religious history and global religious frequency scores in Table 2) are quite similar and therefore no attention was given to these associations since these findings can most likely be interpreted as spurious statistical findings.

Regarding religious involvement throughout childhood and adolescence, in the three age groups analysed, three main issues drew attention: (a) the association with lower severity of crack cocaine consumption (less likelihood of consumption onset before 18 years of age and less craving) and a consistent perception of better quality of life in the psychological domain; (b) more than religious participation, frequency of attendance at religious services was associated with less chance of consumption onset before 18 years of age and lower consumption intensity (less chance of consuming >10 crack cocaine rocks at a single time during the period of greatest consumption) and (c) the frequency of attendance at religious services between 15–17 years was associated with all variables of current consumption severity, and this association was partially mediated by greater participation in social activities (sports and cultural) and the quality of relationship with the parents.

The effects are even clearer when the groups with the highest and lowest religious involvement during childhood and adolescence are compared. The quartile with the lowest attendance at religious services were three times more likely to start crack use before 18 years of age and twice as likely to consume >10 crack cocaine rocks at a single time compared with the quartile of greater frequency.

Our data consistently indicate that religiosity and spirituality provide some protection against drug consumption patterns and imprisonment, and a better quality of life, even in heavy users of crack cocaine. However, this same protection did not occur in relation to criminality, sexual risk-seeking behaviour and psychiatric comorbidities. A possible explanation for these protective findings might be that the discouragement of drug use by the religious community is internalised by children and adolescents and has an effect later in life.^15^ A religious upbringing during childhood may involve being educated with more rules and moral standards, a more structured socio-familiar environment and having internalised values that give meaning to life.^16^ A qualitative study found that family structure and religiosity were the most important factors for reduced drug consumption among young individuals living in a Brazilian shantytown.^32^ The data from two waves of the Add Health study showed that American adolescents who had stated that religion was important to them exhibited a better quality of parent-child relationship and overall family satisfaction.^15^ A USA-based national survey with 36 370 individuals found an association between religiosity and spirituality and a decreased likelihood of drug use disorder, but this association was not substantially mediated by social support or mental health status.^33^ A retrospective study of 1560 twin adult males found that the main protective factors for alcohol use during adolescence were parental monitoring and regular involvement in religious and social activities.^34^

Another notable finding was that, compared with the other age groups, religious involvement between the ages of 15 and 17 years had the strongest association with all variables of later crack cocaine consumption. A meta-analytic review found that religiosity and spirituality had a greater association with the decline of risk-seeking behaviours (use and misuse of alcohol and other drugs, sexual activity) in young adults (18–25 years) than in adolescents (12–17 years).^35^ A possible explanation is that older adolescents are at greater risk of contact with drug users and related risk-seeking behaviours; therefore, being part of a religious social circle in this age group may have a greater protective role.

### Limitations

With respect to the limitations of the study, there may be memory bias relating to the recollection of events that occurred during childhood and adolescence because this was a cross-sectional study. In addition, it is worth noting that the study was conducted in therapeutic communities, which have a strong religious and spiritual component.^18^ Individuals might have been influenced to respond positively to the questions about religiosity and spirituality, thus increasing the percentage of religious individuals. However, such limitations would generate, respectively, a random error of classification and an overestimation of religiosity (even among non-religious individuals), both tending to cancel the possible effects of religiosity and spirituality. Therefore, despite these limitations, we found statistically significant associations that, in reality, may be even stronger. Other methodological limitations also included the arbitrary definition of the set of age periods and the criteria used to convert some multiple-choice answers into data-sets.

### Implications of the study

Even in individuals who were heavy users of crack cocaine, in whom the protective factors of religiosity and spirituality might reasonably have been expected to be ineffective or fail, we found that religious involvement during childhood and adolescence was associated with a less severe crack cocaine consumption pattern, starting use later, lower use intensity and less severe craving symptoms. Our data showed that this association was more important for religious involvement between the ages of 15 and 17 years, an observation that could be highly relevant when considering the design of future prevention strategies. New studies should be undertaken to see if our findings are reproduced in other populations and to investigate the mechanisms of these protective effects.
